# Abnormal Vacuole Membrane Protein-1 Expression in Parkinson’s Disease Patients

**DOI:** 10.3389/fnins.2022.760932

**Published:** 2022-04-06

**Authors:** Murad Al-Nusaif, Cheng Cheng, Tianbai Li, Congcong Jia, Panpan Wang, Song Li, Weidong Le

**Affiliations:** ^1^Center for Clinical Research on Neurological Diseases, First Affiliated Hospital, Dalian Medical University, Dalian, China; ^2^Liaoning Provincial Key Laboratory for Research on the Pathogenic Mechanisms of Neurological Diseases, First Affiliated Hospital, Dalian Medical University, Dalian, China; ^3^Institute of Neurology, Sichuan Academy of Medical Sciences and Sichuan Provincial People’s Hospital, Chengdu, China

**Keywords:** *VMP1* gene, autophagy, Parkinson’s disease, peripheral blood mononuclear cells, biomarker

## Abstract

**Background:**

Parkinson’s disease (PD) is pathologically characterized by progressive dopaminergic (DAergic) neuron loss in the substantia nigra pars compacta (SNpc) and accumulation of intracytoplasmic α-synuclein-containing Lewy bodies. Autophagy has been identified as a critical component in the development and progression of PD. Several autophagy genes have been identified as being altered in PD. One of those genes, vacuole membrane protein-1 (VMP1), an autophagy protein localized in the endoplasmic reticulum (ER) in DAergic neurons, has been shown to cause motor disorder, severe loss of DAergic neurons, and autophagy flux disturbance in the *VMP1* knockout mouse model.

**Objective:**

To evaluate for the first time the alteration on the expression of the VMP1 gene and its clinical correlations in peripheral blood mononuclear cells (PBMCs) of a relatively large sample of PD patients.

**Methods:**

We assessed the *VMP1* mRNA levels in PD patients (*n* = 229) and healthy controls (HC) (*n* = 209) using real-time quantitative PCR (RT-qPCR), and the VMP1 protein levels in PD patients (*n* = 27) and HC (*n* = 27) using Western blot (WB). Then, we analyzed the VMP1 expression levels and clinical features of PD patients.

**Results:**

Our findings revealed that VMP1 levels in the PD group were significantly lower than in the HC group (RT-qPCR *p* < 0.01 and WB *p* < 0.001). The VMP1 expression was significantly lower as the disease progressed, which could be ameliorated by administering DAergic receptor agonists. Moreover, receiver operating characteristic (ROC) curve analysis showed that VMP1 mRNA and protein level area under the curves (AUCs) were 64.5%, *p* < 0.01, and 83.4%, *p* < 0.01, respectively.

**Conclusion:**

This case-control study demonstrates that peripheral VMP1 level altered in PD patients and may serve as a potential endogenous diagnostic marker of PD.

## Introduction

Parkinson’s disease (PD) is the most prevalent movement disorder, primarily as a result of dopaminergic (DAergic) neuron loss in the substantia nigra pars compacta (SNpc), affecting 1% of population over the age of 60 years (Dauer and Przedborski, 2003; Reeve et al., 2014). In genetically predisposed people, environmental variables are assumed to contribute to this complicated and multifaceted disease (Limphaibool et al., 2018).

Pathologically, PD is characterized by an accumulation of aggregation-prone proteins and a loss of protein homeostasis (Proteostasis), culminating in the progressive death of DAergic neurons (Krisko and Radman, 2019). The main neuropathological hallmarks of PD are a substantial loss of DAergic neurons within SNpc and the development of intracytoplasmic α-synuclein-containing Lewy bodies (Hatano et al., 2016; Tysnes and Storstein, 2017). As one of the primary degradation and clearance pathways, autophagy plays a critical role in sustaining effective turnover of the intracellular proteins and damaged organelles (Metaxakis et al., 2018; Wu et al., 2019; Aman et al., 2021). Autophagy can be divided into macroautophagy, microautophagy, and chaperone-mediated autophagy, leading to cargo delivery to the lysosome for degradation and recycling (Parzych and Klionsky, 2014). When autophagy is inhibited, substance clearance is compromised; on the other hand, autophagy stimulation may improve toxic protein clearance (Ghavami et al., 2014; Limanaqi et al., 2020).

The essential roles of the endoplasmic reticulum (ER) appear to be crucial throughout the first stage of autophagy, and one recent study has shown that autophagosome formation is highly related to the ER (Yamamoto and Noda, 2020). Many autophagy-related genes have been correlated to PD, including α-synuclein (SNCA), GBA, LRRK2, ATP13A2, PINK1, PARKIN, VPS35, and FBXO7 (Beilina and Cookson, 2016; Hou et al., 2020). Vacuole membrane protein-1 (VMP1) has been found to be essential in autophagy and membrane trafficking (Vaccaro et al., 2008; Tenenboim et al., 2014). VMP1 is a multi-spanning membrane protein found in the ER that may signal the development of autophagosomes (Ropolo et al., 2007; Vaccaro et al., 2008). Its location has been identified in the Golgi apparatus, the ER (Dusetti et al., 2002; Calvo-Garrido et al., 2008), autophagosomes (Ropolo et al., 2020), and the plasma membrane (Sauermann et al., 2008). Recent studies found that VMP1 has a highly dynamic performance with lipid droplets (LD), endosomes, and mitochondria (Tábara and Escalante, 2016). In addition to the contacts between the ER and other organelles, more recent research has revealed that VMP1 regulates the connection between the ER and the isolation membrane (Zhao et al., 2017).

The *VMP1* gene encodes a transmembrane protein of around 400 amino acids, such as autophagy-related protein (ATG) (Dusetti et al., 2002). When the ATG domain of VMP1 is altered, it loses its capacity to attract microtubule-associated protein 1 light chain 3 (LC3) and its interaction with Beclin-1, preventing autophagy initiation (Kocak et al., 2021). The depletion of *VMP1* increases the number of contacts between ER and isolation membrane, mitochondria, LD, and endosomes. Furthermore, this depletion limits the dynamicity of the connections, resulting in spherical and inflated mitochondria (Wang et al., 2020). More interesting is that under suboptimal dietary conditions, recent research found that depleting *VMP1* stimulates LC3 formations in ER of mammalian cells with depleted *VMP1* (Tábara and Escalante, 2016). Thus, *VMP1* may be engaged in the autophagy pathway by altering the ER and organelle contacts. These results suggest that a *VMP1* deficit affects autophagosome development, disrupts interaction with the ER, and precludes fusion with the lysosome (Zhao et al., 2017). Many additional studies in *Dictyostelium*, *Arabidopsis thaliana*, and *Chlamydomonas* revealed that *VMP1* is involved in various cellular processes such as cytokinesis, phagocytosis, osmoregulation, and protein secretion (Zhao et al., 2017; Tábara et al., 2019).

Our recent study, using a mouse model of selective *VMP1* conditional knockout (cKO) in DAergic neurons, found that *VMP1* deficient in DAergic neurons caused motor disorders, significant DAergic neuronal loss as well as autophagy flux disruption. The increase in α-synuclein accumulation was found in the striatal of these *VMP1* cKO mice, indicating that VMP1 may play a pivotal role in preventing misfolded protein-induced DAergic neuronal loss. Also, we found that VMP1 influences neural longevity survival and axonal homeostasis, which may play a role in the preclinical model to explain the mechanism of DAergic neuronal loss (Wang et al., 2021). Therefore, the *VMP1* gene might have an essential role in the pathological abnormalities of PD patients. The purpose of the study would be to determine whether there are any differences in VMP1 levels in peripheral blood mononuclear cells (PBMCs) from PD patients and healthy controls (HC). Additionally, to investigate the potential association between VMP1 expression and the clinical characteristics of patients with PD.

## Materials and Methods

### Participants

A total of 492 participants, 256 patients with idiopathic PD, and 236 HC were enrolled in this study between January 2015 and July 2021 (Table 1). The male/female ratio was at 262/230. The age of the participants ranged from 41 to 90, with an average of 66.77 years. Patients with PD in this study were recruited from those who visited the Neurology Department of the First Affiliated Hospital of Dalian Medical University. The PD patients were diagnosed as idiopathic PD according to the Movement Disorder Society Clinical Diagnostic Criteria, and all secondary parkinsonism was not included in this study (Postuma et al., 2015). The Modified Hoehn and Yahr (M-HY) staging assessed the PD motor symptoms (Goetz et al., 2004). Participants of the HC group were all from the Health Examination Center of the First Hospital of Dalian Medical University and demonstrated that they did not have any apparent neurological or non-neurological disorders.

**TABLE 1:** Demographics and clinical characteristics of PD patients and HC.

	RT-qPCR study groups				WB study groups			
Characteristics	PD patients (*n* = 229) (52%)	*p*-value	HC (*n* = 209) (48%)	*p*-value	PD patients (*n* = 27) (50%)	*p*-value	HC (*n* = 27) (50%)	*p*-value
Age	(41–88) years 67.12 ± 88*^a^*	NS	(45–90) years 66.27 ± 44*^a^*	NS	(52–78) years 66.22 ± 92*^a^*	NS	(59–86) years 66.5 ± 93*^a^*	NS
Gender*^b^*								
M	121 (53%)	NS	116 (56%)	NS	12 (44%)	NS	13 (48%)	NS
F	108 (47%)		93 (44%)		15 (56%)		14 (52%)	
Disease duration (years)	5.81 ± 5*^a^*	–	NA	–	6.28 ± 4*^a^*	–	NA	–
1–5 years	113 (49%)				15 (56%)			
6–10 years	92 (40%)				9 (33%)			
11–20 years	31 (11%)				3 (11%)			
M-Hoehn and Yahr scale (M-HY)	2.28 ± 6*^a^*	–	NA	–	2.31 ± 48*^a^*	–	NA	–
1–1.5	40 (17%)				6 (22%)			
2	71 (31%)				7 (26%)			
2.5–3	104 (45%)				12 (44%)			
4–5	14 (6%)				3 (11%)			
Not treated with anti-Parkinson’s drugs	70*^c^*	–	NA	–	11*^d^*	–	NA	–

*PD, Parkinson’s disease; HC, healthy control; M, male; F, female; NS, not significant; NA, not analyzed; SD, standard deviation; RT-qPCR, real-time quantitative PCR; WB, Western blot. ^a^Data are expressed as mean ± SD. ^b^Indicates generated by the Chi-square test. ^c^Fifty-eight on L-dopa monotherapy, 24 on DA receptor (DR) agonist monotherapy, 68 on a combination of DR agonist and L-dopa and 9 treated irregularly with various other medications. ^d^Five on L-dopa monotherapy, and 11 on a combination of DR agonist and L-dopa.*

### Standard Protocols Validation, Recordings, and Patient Permissions

The study has been granted ethical approval by the First Affiliated Hospital with Dalian Medical University Ethics Committee (approval number: LCKY2014-29). All participants signed a written informed consent form.

### Blood Sampling and Peripheral Blood Mononuclear Cells Separation

The peripheral blood sample was collected from the cubital vein in a collection tube containing ethylenediaminetetraacetic acid (EDTA). The PBMCs were then separated from the peripheral blood in lymphocyte separation medium (HAOYANG, Tianjin, China), centrifuged at 450 *g* for 20 min, washed twice, then red blood cell lysis buffer was added, centrifuged at 300 *g* for 10 min, and washed once. Samples are either used immediately or frozen at −80°C for later use.

### mRNA Extraction and Quantitation From Peripheral Blood Mononuclear Cells

Total mRNA was extracted from PBMCs using the mRNA™ Isolation Kit (AMBION, Carlsbad, CA, United States). The ABI 7500 fast real-time PCR instrument (Applied Biosystems, Foster City, CA, United States) measures the mRNA level of *VMP1* in PBMCs using quantitative real-time polymerase chain reaction (qRT-PCR). The specific primers targeting PBMCs are presented in Supplementary Table 1. After 2 min at 95°C, the reaction was carried out at 95°C for 40 cycles for 10 s and at 60°C for 32 s, with the PCR product Hieff UNICON^®^ Universal Blue qPCR SYBR Green Mater Mix (Shanghai Yisheng Biotechnology China Shanghai-Co., Ltd.). Furthermore, quantitative fluorescence analysis was determined by the difference in Ct (ΔCt) between the *VMP1* gene and the internal control gene *GAPDH*, and the relative expression level of *VMP1* was calculated using the 2^–Δ*Ct*^ method.

### Protein Extraction and Quantitation From Peripheral Blood Mononuclear Cells

Considering the functions of autophagy in the degradation and elimination of abnormal or aggregated proteins, we further examined and compared the expressions of VMP1 protein in the PD and HC groups. RIPA lysis buffer (Beyotime, China) plus the protease inhibitor cocktail (100:1, Sigma-Aldrich, United States) was used to resuspend PBMCs. After 4 s of homogenization, the samples were put on ice for 30 min before centrifuging them at 12,000 × *g* for 15 min. We measured the protein concentration in the supernatant using the BCA Protein Assay Kit (Beyotime, China). After mixing with the loading buffer and boiling at 95°C for 5 min, equal amounts of protein extracted from the PBMCs (HC and PD groups) were subjected to Western blot (WB) analysis and electrophoretic separation by 10 or 12.5% SDS-PAGE. After that, the protein was transferred to a 0.45 μm PVDF membrane (Millipore, Burlington, MA, United States). The blots were then blocked in TBS-T with 5% skimmed dry milk at room temperature for one hour and then immuno-probing with antibodies overnight at 4°C. The membranes were incubated with anti-VMP1 and anti-GAPDH antibodies. Information about the particular antibodies used in the present study was summarized in Supplementary Table 1. An anti-rabbit secondary antibody was added after washing with Tris-buffered saline-Tween (TBST) 3 times (10 min each time). Incubate the membrane at 37°C for 1 hour, and then wash 3 times with TBST (10 min each). A chemiluminescence detection kit (Wan Lei Biotechnology, Shenyang, China) was used to identify protein bands. The FluorChem Q system (Protein Simple, San Jose, CA, United States) was used to measure the amount of chemiluminescence and was normalized based on the value of the reference protein.

### Statistical Analysis

The Shapiro–Wilk test was used to evaluate the data normality. A Chi-square test was used to evaluate the statistical difference in gender between the PD and HC subjects. The Student’s *t*-test was used to analyze differences between two groups in the age distribution. The mean ± standard error (SE) represents the quantitative data of each group. The Mann–Whitney U-test analyzed differences between the PD and HC subjects for VMP1 mRNA and protein expression. A non-parametric one-way analysis of variance analysis (ANOVA), was performed using the GraphPad Prism software version 6 (GraphPad Inc., San Diego, CA, United States) to evaluate the differences in the mean value of the mRNA and protein expressions in individuals from each group. The predictive performance of VMP1 mRNA and protein was quantified using a receiver operating characteristic (ROC) curve and the area under the curve (AUC) using version 22.0 of the Statistical Package for Social Science (SPSS software Inc., Chicago, IL, United States). All statistical analyses were carried out on both sides of the tail, and a *p*-value of less than 0.05 was considered statistically significant.

## Results

### Characteristics of the Study Population

A total of 492 participants, all Han ethnic Chinese, 256 patients with idiopathic PD (aged 45–90 years, average 66.67), and 236 HC (aged 41–88 years, average 66.87) were enrolled in this study. In 438 subjects the PD (*n* = 229) and HC (*n* = 209) groups, we measured their *VMP1* mRNA level in PBMCs. In the remaining 54 subjects in the PD (*n* = 27) and HC (*n* = 27) groups, we measured the VMP1 protein expression in PBMCs. The demographic characteristics of PD patients and HC of both mRNA and protein tested groups are summarized in Table 1. After determining the VMP1 expression level, we conducted clinical analysis on the groups included in the real-time quantitative PCR (RT-qPCR) and WB. No significant difference in gender and age was found among the PD and HC groups. This study evaluated the disease duration, M-HY score, and anti-Parkinson drug use status of PD patients (Table 1). Following that, in the results section, we compared the VMP1 expression levels of each group to HC.

### Altered Expression of Vacuole Membrane Protein-1 in the Peripheral Blood Mononuclear Cells of Parkinson’s Disease Compared to Healthy Controls

**FIGURE 1:**
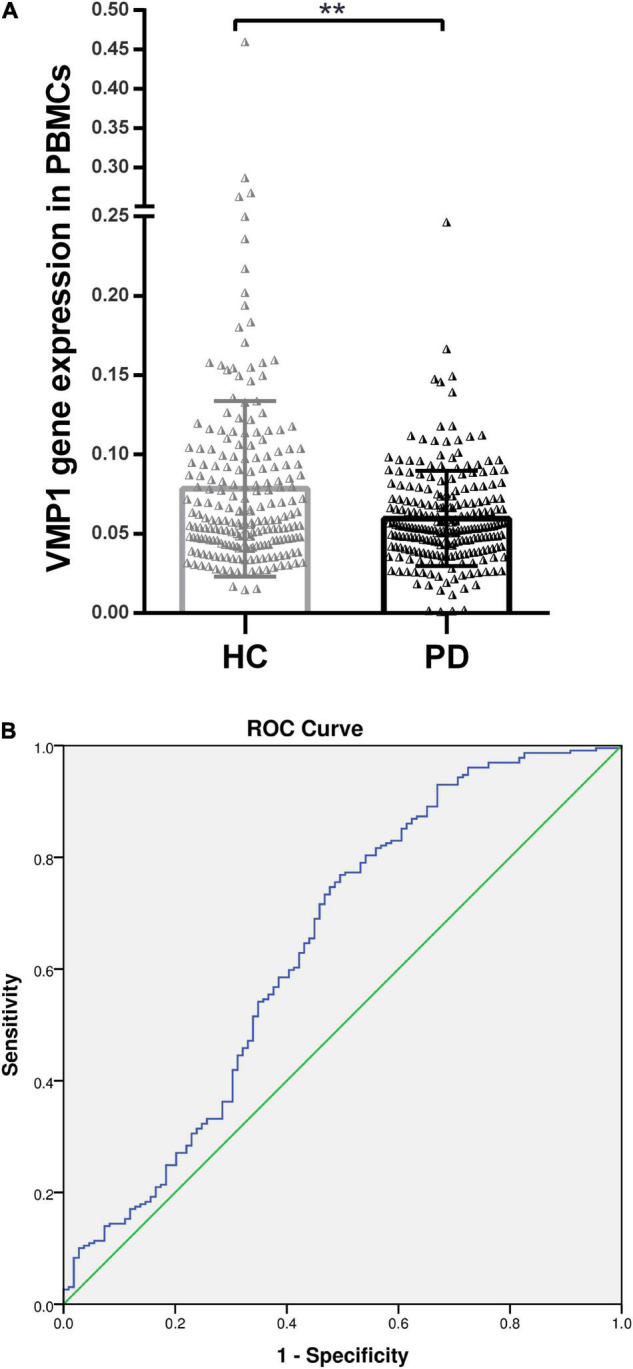
Real-time quantitative PCR analysis for the *VMP1* expression levels in PBMCs. **(A)** Scatter plots of *VMP1* relative mRNA expression level in the PBMCs of HC (*n* = 209) and PD (*n* = 229). Horizontal bars represent mean and SE values. **p < 0.01. **(B)** Receiver operation curve for *VMP1* was (64.5%, 95% CI, 0.57–0.709) *p* < 0.01.

**FIGURE 2:**
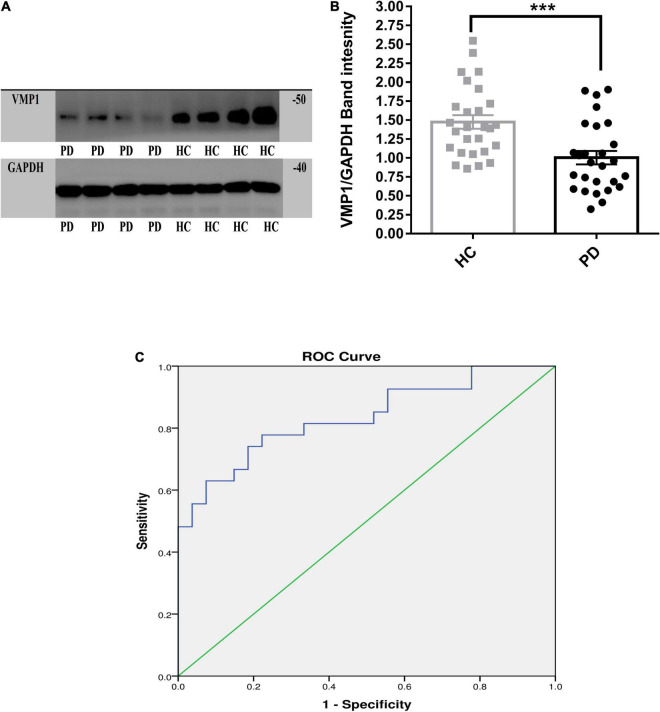
Western blot analysis for the VMP1 expression levels in PBMCs. **(A)** WB analysis for the VMP1 expression levels in PBMCs. **(B)** Quantification of WB analysis of (PD = 27 and HC = 27). **(B)** Analyzed by using *t*-test Mann–Whitney test two-tailed *p*-value. Data were represented as mean ± SEM. ***p < 0.001. **(C)** Receiver operation curve for VMP1 was (83.4%, 95% CI, 0.726–0.942) *p* < 0.01.

We measured VMP1 mRNA and protein levels in PD patients (*n* = 256) and HC (*n* = 236) PBMCs using RT-qPCR and WB. Our data showed that VMP1 levels in the PD group were significantly lower than in the HC group (RT-qPCR *p* < 0.01; Figure 1A), and WB (*p* < 0.001; Figures 2A,B). The correlation between the mRNA and protein VMP1 expression was performed, and we found a positive correlation with a Spearman rank coefficient (*r*_*s*_ = 0.40) *p* < 0.03 and 95% confidence interval (0.145–0.685).

### The Performance Expression Level of the Vacuole Membrane Protein-1 for Parkinson’s Disease Patients

We investigate the relevance of VMP1 expression in PBMCs for PD Patients by AUC using ROC curve analysis. The AUC of *VMP1* in mRNA was (64.5%, 95% CI, 0.57–0.709) *p* < 0.01 (Figure 1B) and in the protein level was (83.4%, 95% CI, 0.726–0.942) *p* < 0.01 (Figure 2C).

### Associations Between the mRNA and Protein Expression Levels of the Vacuole Membrane Protein-1 Gene With the Clinical Characteristics of Parkinson’s Disease Patients

#### The Influence of the Disease Duration and Severity on the Vacuole Membrane Protein-1 Expression in Parkinson’s Disease

**FIGURE 3:**
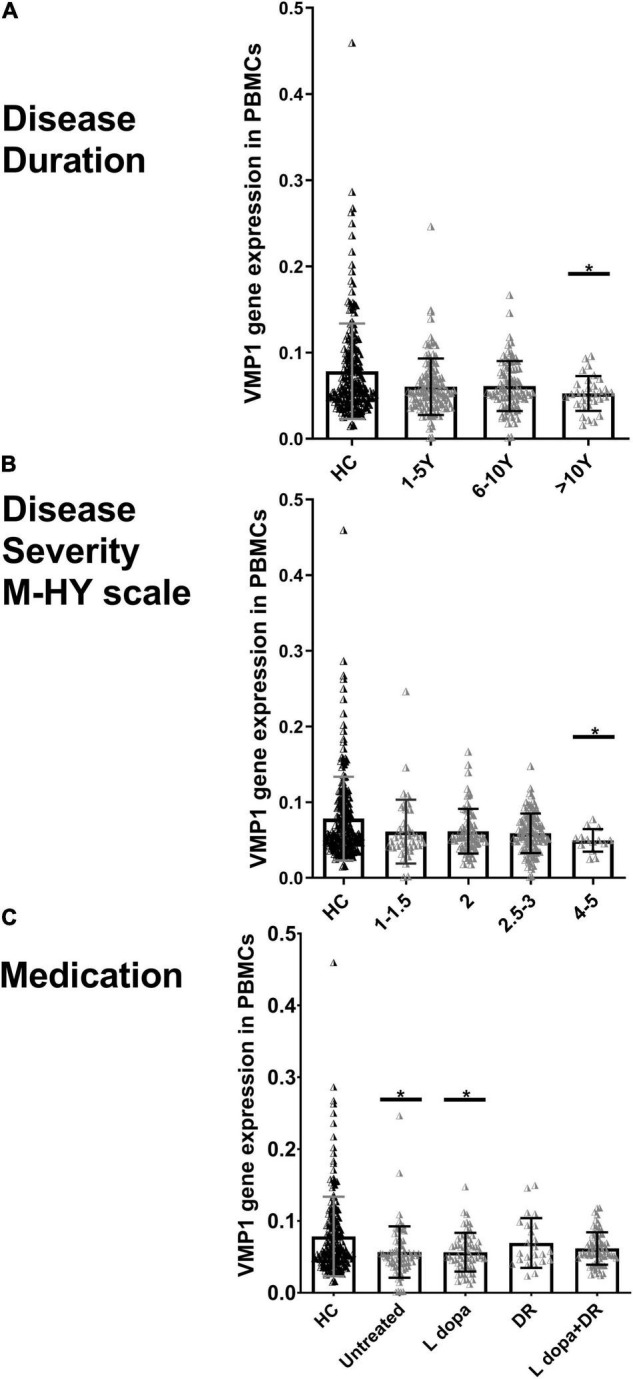
Real-time quantitative PCR analysis for the *VMP1* expression levels in PBMCs and clinical characteristics of PD patients. **(A)** Disease duration, **(B)** disease severity M-HY, Modified Hoehn and Yahr scale, and **(C)** medications. DR, DA receptor agonists monotherapy; L-dopa, L-dopa monotherapy; L-dopa + DR, the combination of DA agonists and L-dopa. Horizontal bars represent mean and SE values. *p < 0.05 significantly lower than that of HC.

We explored the association between the disease duration (the number of years from the start of symptoms) and the severity (M-HY scale) in PD patients and the VMP1 expression in both mRNAs (*n* = 229) and protein (*n* = 27) samples. The disease duration was divided into three groups: 1–5 years (*n* = 113), 6–10 years (*n* = 92), and 11–20 years (*n* = 31). Our results revealed that the level of *VMP1* in mRNA was low in all three groups and showed a marked decrease during the late-stage of disease progression than in the early stage of disease (Figure 3A). As for protein levels, 1–5 years (*n* = 15), 6–10 years (*n* = 9), and 11–20 years (*n* = 3), the VMP1 level was reduced in all three groups and significantly reduced expression in the 1–5 years and 6–10 years groups (*p* < 0.05 and *p* < 0.01), but not in the 11–20 years group. In aspects of severity score, our findings revealed that the level of *VMP1* in mRNA was low in all five groups and showed a significant decrease in expression as disease severity increased (Figure 3B). In terms of protein expression, it decreased in all PD patients and decreased more as disease severity increased. A total of 2, 2.5, 3, and 4–5 M-HY showed a significantly reduced expression (*p* < 0.05). The severity section showed that VMP1 levels were somewhat higher in the 2.5–3 group compared to the 2M-HY and 4–5 M-HY groups. One possible explanation is using DA receptor (DR) agonists or DR agonists plus L-dopa. We performed a group analysis based on treatment type and discovered that the untreated and L-dopa monotherapy groups of 2.5–3 M-HY had reduced VMP1 expression compared to the DR agonist and DR agonists plus L-dopa groups in the same group. This will be discussed more in the section on medicine and VMP1 expression.

#### The Influence of Medications on Vacuole Membrane Protein-1 Expression

In addition, we grouped PD patients into four groups depending on whether or not they were taking medication. In the mRNA analysis of 229 PD patients, 70 were not treated with anti-Parkinson drugs. In contrast, the rest were treated with anti-Parkinson medications, including 58 on L-dopa monotherapy, 24 on DR agonist monotherapy, and 68 on a combination of DR agonist and L-dopa. In the four groups, *VMP1* expression decreased and reached a significant level in both the untreated and L-dopa monotherapy groups *p* < 0.05 (Figure 3C). Nine PD patients were excluded who were being treated irregularly with a variety of other medications. In the protein level experiments, 11 people did not use anti-Parkinson drugs, 5 people were on L-dopa alone, and 11 people were on a combination of DA agonists and L-dopa. VMP1 levels were significantly lower in the untreated and L-dopa monotherapy PD groups *p* < 0.01 but not in the other groups.

## Discussion

In this study, we measured the mRNA and protein expression of VMP1 in the PBMCs of PD and HC simultaneously. Following that, we analyzed the alteration in the VMP1 expression of PD with the disease duration, M-HY score, and anti-Parkinson drug use status. The main finding is that mRNA, and protein VMP1 expression decreased in PD and positively correlated, which could be a potential marker for autophagy alteration in PD patients. Studies assessed autophagy markers in idiopathic PD patients’ PBMCs compared to human individuals, indicating autophagy alteration in PD patients (Papagiannakis et al., 2015; Miki et al., 2018). As we stated above, the protein aggregation in PD was yielded by increased misfolded protein and a malfunction in the ubiquitin-proteasome and/or autophagic/lysosomal pathways (Vilchez et al., 2014; Pitcairn et al., 2019). VMP1 is required for autophagosome and lysosome fusion as well as maintaining interaction with the ER. Moreover, our recent result found that a selective *VMP1* cKO in DAergic neuron mice results in motor deficits, severe DAergic neuron loss, mitochondrial abnormalities, and an interruption of autophagy flux in the SNpc neurons (Wang et al., 2021). The change in autophagosome trafficking appears to be consistent with that seen in *VMP1*-depleted cells, which is intriguing because VMP1 depletion impairs autophagosome maturation and dissociation from the ER, failing to fuse with the lysosome (Molejon et al., 2013; Zhao et al., 2017). Consequently, alteration in autophagy could be the mechanism attributed to the VMP1 expression decrease in PD patients.

According to our results, the expression level of VMP1 in PBMCs’ mRNA and protein levels of PD patients decreased with the disease progression in both duration and severity. The aggregation of misfolded proteins drives the progression of many neurodegenerative diseases. A recent study showed that vesicle trafficking is critical in PD and may contribute to disease severity (Gao et al., 2021). As a result, autophagic activity is thought to influence disease severity to some extent. The amount of α-synuclein in DAergic neurons is an important determinant of neurotoxicity in PD; therefore, effective α-synuclein clearance is an essential determinant of PD severity (Winslow et al., 2010). Rott et al. (2011) indicate that deubiquitinated α-synuclein is the primary target of autophagy and that degradation of α-synuclein was critical in a transgenic mouse model (Wu et al., 2016). In contrast, inclusions containing α-synuclein were shown to reduce autophagic activity during the maturation of autophagosomes and their fusion with lysosomes (Button et al., 2017). As a result, the effect of α-synuclein aggregation and the autophagy changes as PD progressed could be one reason for the decline in VMP1 expression as disease duration and severity increase. We analyzed the AUC for both mRNA and protein expression of VMP1 levels, which revealed that the AUC value in the ROC curve analysis of mRNA was (64.5%) *p* < 0.01 and at the protein level was (83.4%) *p* < 0.01. There is also a positive correlation between mRNA and protein expression levels. These suggest that the PBMCs VMP1 could be a potential biomarker for PD patients. Identifying biomarkers in PD patients’ blood may help assess the disease’s severity and progression (Yang et al., 2020).

Vacuole membrane protein-1 levels were significantly reduced in the untreated and L-dopa monotherapy PD patients, but not in those on DR agonist monotherapy or a combination of DR agonist and L-dopa. A possible explanation is that the DA agonist may potentiate the autophagy effect of VMP1. Previous cell and animal model research indicate that pramipexole, a DRD2 and DRD3 agonist, is a potent inducer of autophagy in both cell and animal models (Barroso-Chinea et al., 2020). Further research is still needed to understand the mechanism and role of DA agonists in the VMP1.

There are limitations to this study that should be considered. First, because of the high variability of the factors’ effects on autophagy, starvation, nutrition, comorbidities, and other factors were not included, affecting VMP1 expression, which may need subjective and objective assessment methods to utilize a retrospective questionnaire, which may be influenced by recall bias. Second, there is a lack of preclinical studies about the effect of VMP1 on PD duration, severity, and therapy models. Finally, our study was limited to a single location and ethnic group. It may be interesting to determine the diverse groups of people to obtain a more representative picture.

## Conclusion

Compared with HC, the expression of the *VMP1* gene in PD patients was significantly reduced. Our previous preclinical findings, together with this clinical data, might give a clue to the importance of VMP1 and the lower pathway. The expression of VMP1 was significantly lower as the disease progressed, which could be used as biomarkers to aid PD diagnosis performance. VMP1 expression was low in the untreated and L-dopa-treated groups, but it was near normal in patients with DA agonist therapy, which we think will open a new field of target therapy for treating PD.

## Data Availability Statement

The raw data supporting the conclusions of this article will be made available by the authors, without undue reservation.

## Ethics Statement

The studies involving human participants were reviewed and approved by the Ethics Committee of the First Affiliated Hospital of Dalian Medical University (approval number: LCKY2014-29). The patients/participants provided their written informed consent to participate in this study.

## Author Contributions

MA-N, CC, and TL: samples collection, methodology, experimental work, writing—original draft, and data analysis. WL: project supervision, administration, methodology, and manuscript editing. CJ, PW, and SL: data curation, resources, formal analysis, validation, and revising the manuscript. All authors contributed substantially to this work and approved the final manuscript.

## Conflict of Interest

The authors declare that the research was conducted in the absence of any commercial or financial relationships that could be construed as a potential conflict of interest.

## Publisher’s Note

All claims expressed in this article are solely those of the authors and do not necessarily represent those of their affiliated organizations, or those of the publisher, the editors and the reviewers. Any product that may be evaluated in this article, or claim that may be made by its manufacturer, is not guaranteed or endorsed by the publisher.
