# Supplementary material for: Abnormal Vacuole Membrane Protein-1 Expression in Parkinson’s Disease Patients
Source: Front Neurosci. 2022 Apr 6;16:760932. doi: 10.3389/fnins.2022.760932 (PMC9019220; doi:10.3389/fnins.2022.760932)
Supplement: Supplementary file 1 [file Table_1.DOCX]

**Supplementary Table S1.** List of primers and Antibody used for quantitative real-time PCR assays and WB respectively

| mRNA | Primers Sequence (5′–>3′) |
| --- | --- |
| Vmp1 | Forward: (5′–TTTCCCGAACCACCCTATCC -3′) |
|  | Reverse: (5′–CAGACTCTGCATGTTCCAGC- 3′) |
| GAPDH | Forward: (5′–GCGGTCACGTTTTCCACTATG- 3′) |
|  | Reverse: (5′–GAAGATGGTGATGGGATTTC- 3′) |

| Target | Species | Application | Dilution | Company | Cat. number |
| --- | --- | --- | --- | --- | --- |
| VMP1 | Rabbit | WB | 1:1000 | CST | 12929S |
| GAPDH | Rabbit | WB | 1:2000 | CST | 2118 |
